# Supplementary material for: Pupillary manifolds: uncovering the latent geometrical structures behind phasic changes in pupil size
Source: Sci Rep. 2024 Nov 8;14:27306. doi: 10.1038/s41598-024-78772-x (PMC11549318; doi:10.1038/s41598-024-78772-x)
Supplement: Supplementary file 1 — Supplementary Material 1 [file 41598_2024_78772_MOESM1_ESM.pdf]

Supplementary material for

Pupillary manifolds: uncovering the latent geometrical structures behind phasic changes in pupil size

***Elvio Blini, Roberto Arrighi, and Giovanni Anobile**

Department of Neuroscience, Psychology, Pharmacology and Child Health, University of Florence, Florence, Italy

*** Corresponding author:**

Elvio Blini

elvioadalberto.blini (at) unifi.it

Firenze, 50136, Via di San Salvi 12, Building 26

Supplementary figures captions

Figure S1: phasic pupil size changes are intrinsically low-dimensional. The figure depicts the scree plots from principal component analysis on the three tasks. Three components are generally sufficient to explain more than 88% of the overall variability in the generating data. Components above 3 account for less than 3% of the remaining variance.

Figure S2: behavioral performance in the WML mapping task. Mean (95% confidence interval) accuracy in reporting the presented numbers as a function of cognitive load. Performance was near ceiling up until 4 digits, then decreased rapidly with 5 and, especially, 6 digits.

Figure S3: behavioral performance in the combined task. Mean (95% confidence interval) accuracy in reporting the presented numbers as a function of cognitive load and luminance level. Performance was near ceiling up until 4 digits, then sensibly decreased, regardless of luminance.

Figure S4: fingerprint component of reflexes to light. One rotated component, RC3, obtained from the PLR mapping task, mapped efficiently the entire luminance space on a latent dimension. Values depict mean scores (95% confidence interval).

Figure S5: the fingerprint of reflexes to light do not map cognitive load. One rotated component, RC3, obtained from the WML mapping task, did not map distinct cognitive load conditions. Values depict mean scores (95% confidence interval).

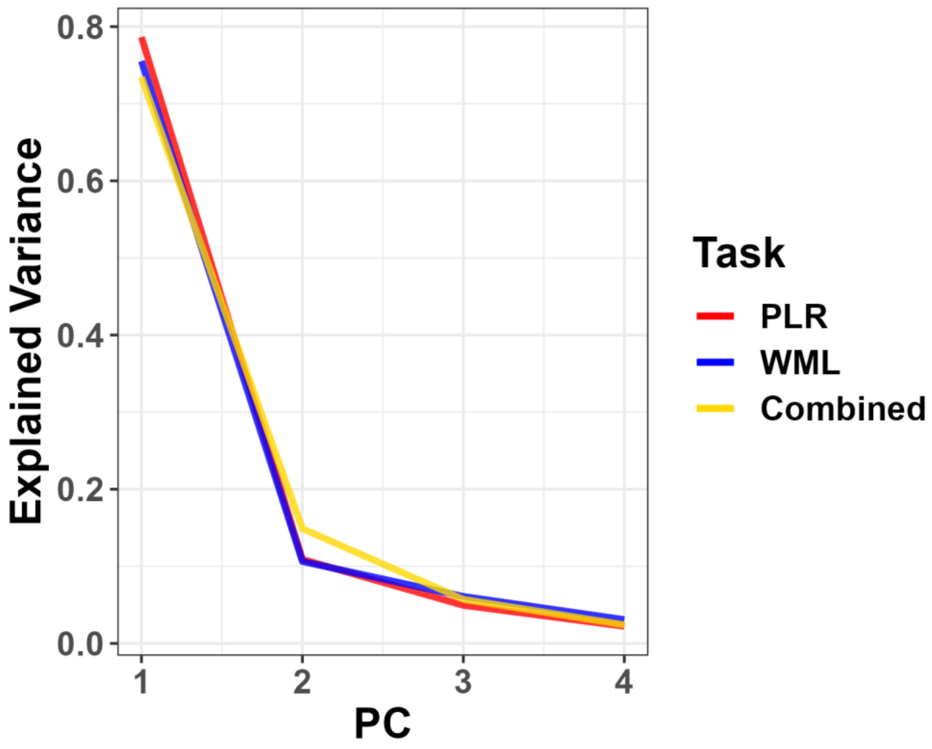
Explained Variance
0.8
0.6
0.4
0.2
0.0
1
2
3
4
PC
Task
PLR
WML
Combined

Figure S1.

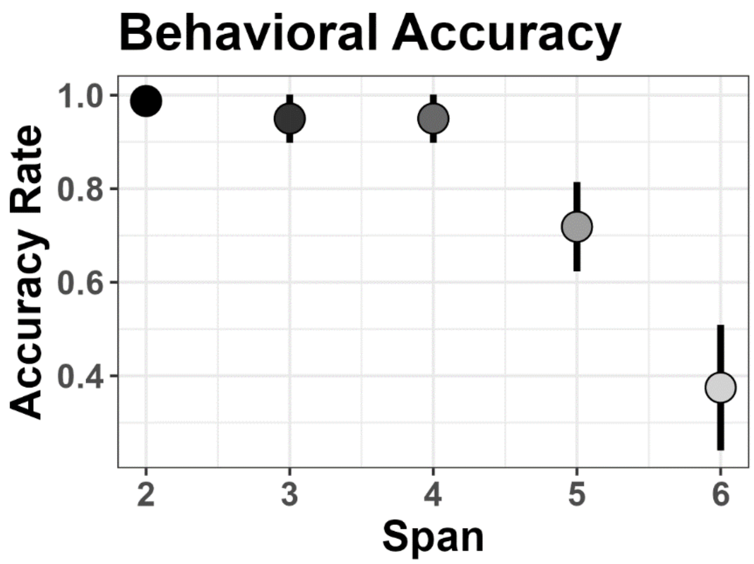

Figure S2.

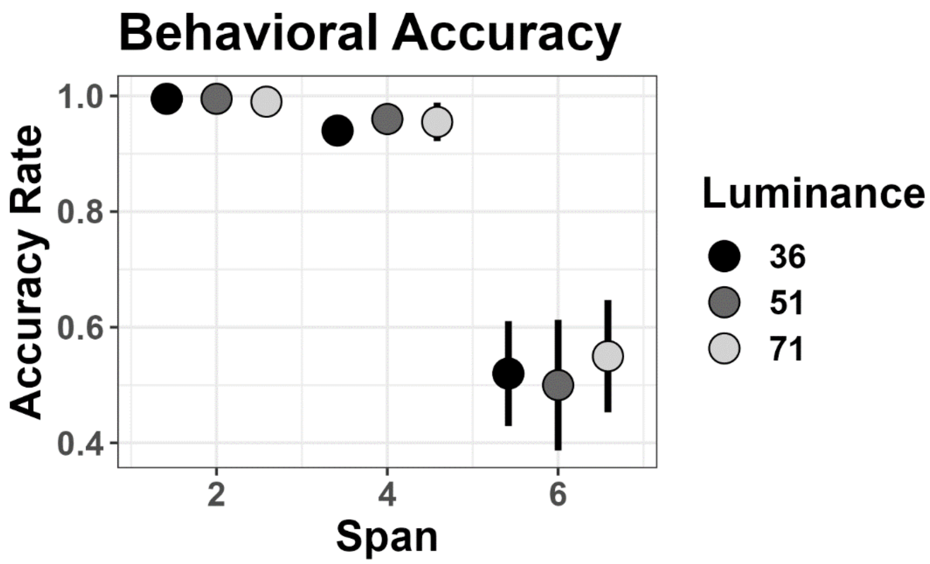
Behavioral Accuracy
Accuracy Rate
1.0
0.8
0.6
0.4
2
4
6
Span
Luminance
36
51
71

Figure S3.

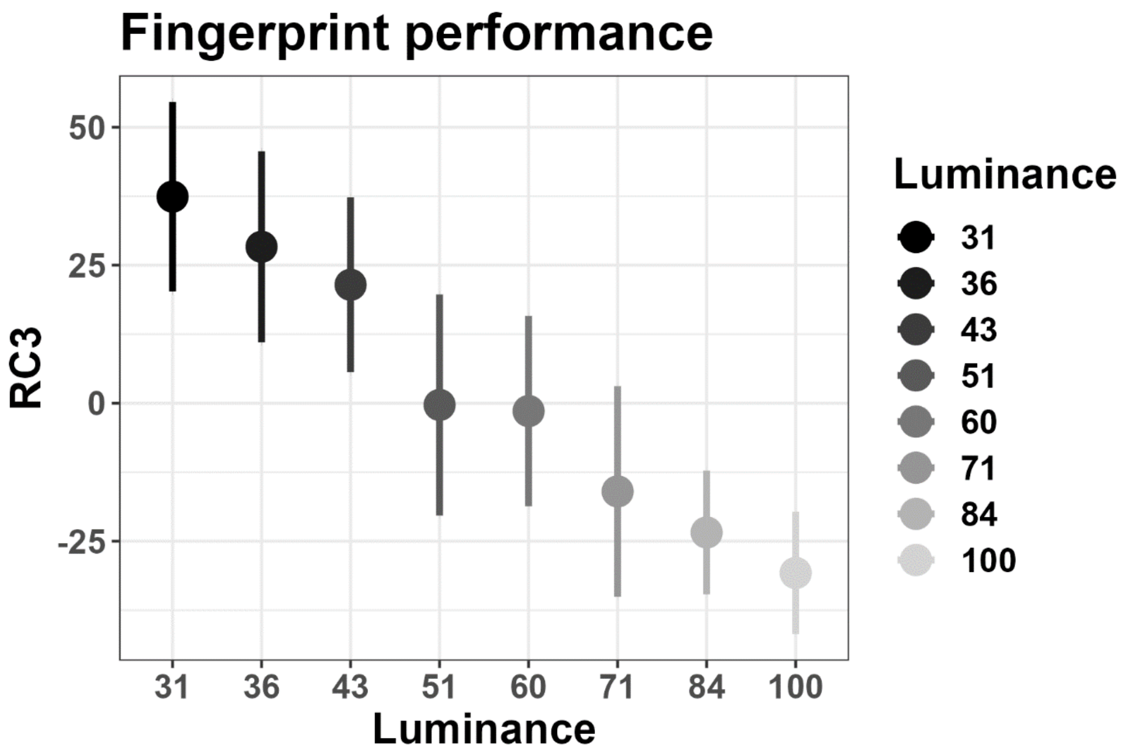
Fingerprint performance
RC3
50
25
0
-25
31
36
43
51
60
71
84
100
Luminance
Luminance
31
36
43
51
60
71
84
100

Figure S4.

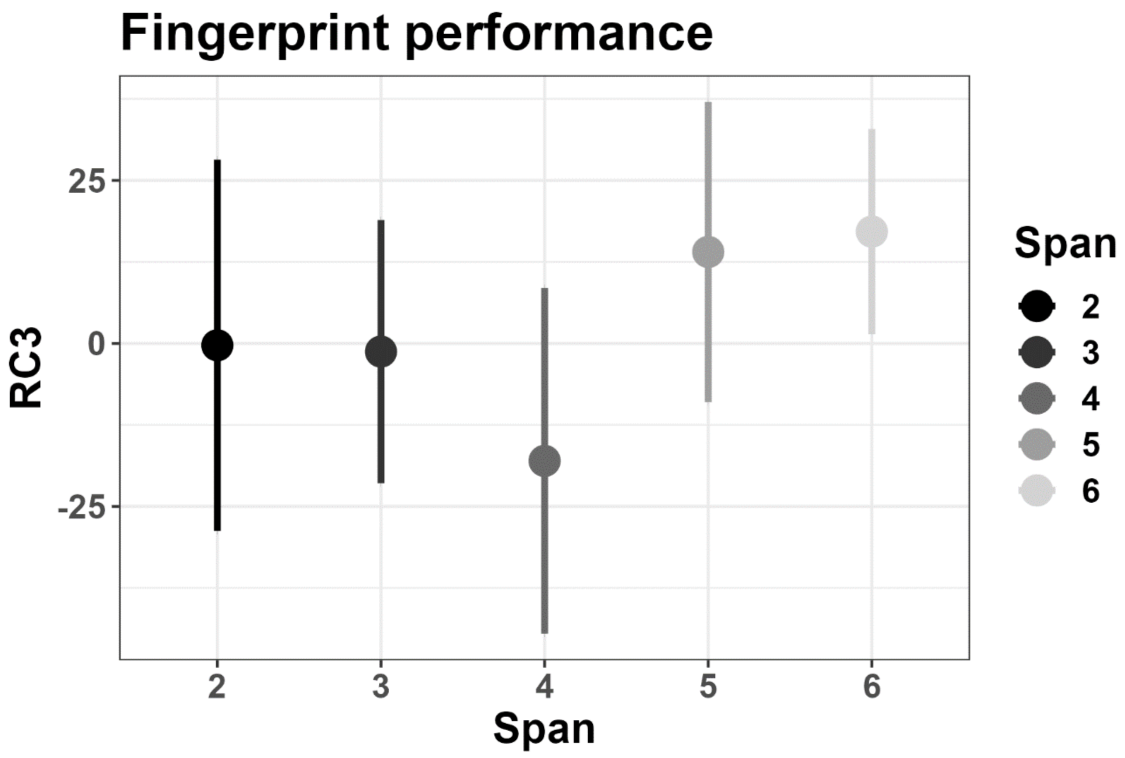

Figure S5.
